# Public health round up

**DOI:** 10.2471/BLT.20.010120

**Published:** 2020-01-01

**Samoa measles outbreak**

A three-year-old Samoan girl is vaccinated against measles as part of the government’s response to an outbreak that was declared in October.

**Figure:**
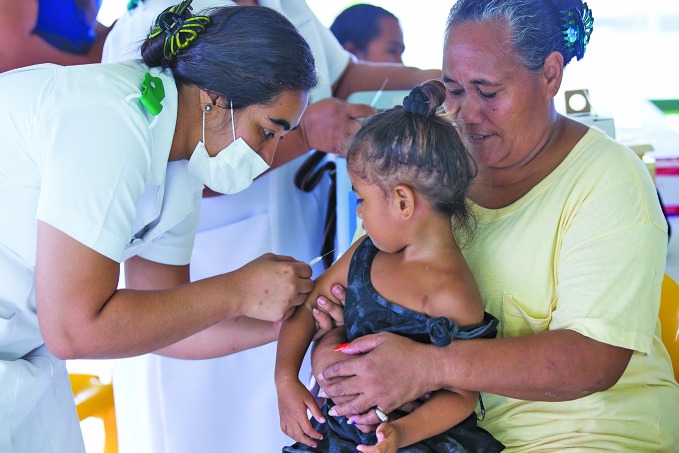
UNICEF / Allan Stephen

## Pacific islands measles

The Samoa Ministry of Health declared a measles outbreak on 16 October and a state of emergency on 15 November.

As of 4 December, a mandatory measles vaccination programme had been implemented. Schools were temporarily closed and children under 18 years of age stopped from attending public gatherings or going to medical facilities, unless they require immediate medical attention.

The health ministry reported a total of 4052 measles cases since the start of the outbreak, and 60 deaths.

http://bit.ly/2RFzJcC

## Surge in measles cases

Worldwide, there were an estimated 9.8 million measles cases in 2018, up from 7.6 million estimated cases in 2017. The estimates were reported in *Progress toward regional measles elimination worldwide*, a joint publication of the World Health Organization (WHO) and the United States Centers for Diseases Control and Prevention (CDC). WHO published the report on 6 December in the *Weekly epidemiological record*.

Estimated measles-related deaths also increased from 124 000 in 2017 to 142 300 deaths in 2018.

One of the reasons for the increase is a failure to achieve optimal vaccination coverage. Vaccination rates globally have stagnated for almost a decade. The World Health Organization (WHO) and the United Nations Children's Fund (UNICEF) estimate that only 86% of children received the first dose of measles vaccine through their country’s routine vaccination services in 2018, while fewer than 70% received the second recommended dose.

http://bit.ly/2RyncYH

## Violence disrupts Ebola response

Violence against people working on the Ebola response in the Democratic Republic of the Congo severely disrupted efforts to contain the Ebola epidemic at the end of 2019.

Targeted attacks took place at the end of November, resulting in the death of four people involved in the Ebola response and the injuring of five others. Widespread civil unrest also prevented workers from getting to communities affected by the Ebola outbreak. 

As a result, fewer alerts were reported and investigated in the first week of December and fewer contacts (people known to have been in contact with infected individuals) were registered and followed compared with the average for November.

According to WHO, there were approximately 390 attacks on health facilities in the Democratic Republic of the Congo in 2019, resulting in 11 deaths and 83 health-care workers and patients injured.

As of 3 December, 3313 people had been infected and 2207 people had died since the outbreak was declared in August 2018.

http://bit.ly/36g7x49

## Plan to tackle HIV drug resistance

WHO and partners launched a five-year regional action plan to monitor, prevent and respond to human immuno-deficiency virus (HIV) drug resistance in Africa.

Developed by WHO, the plan was presented at the International Conference on AIDS and STIs in Africa (ICASA), which took place between 1 and 6 December in Kigali, Rwanda.

Africa is home to 70% of the world’s people living with HIV and 66% of all new infections. Growing resistance to HIV drugs in Africa is threatening the significant progress made in the global fight against the virus.

http://bit.ly/2Pnu20o

## Inadequate climate change response

Most countries are failing to fully implement plans that they developed in response to climate change, according to *2018 WHO health and climate change survey report: tracking global progress.*

The report*,* published on 3 December, draws on data from 101 countries surveyed by WHO in 2018 and provides a snapshot of progress made in the field of health and climate change. According to the report, only half of the countries surveyed have developed a national health and climate change strategy, and only 10% of countries with strategies have committed the resources required for full implementation.

http://bit.ly/2PwYWmW

## Malaria progress challenged

According to WHO’s *World Malaria Report 2019,* which was published on 4 December, cases declined from an estimated 231 million in 2017 to 228 million in 2018. Estimated deaths declined from 416 000 to 405 000 over the same period.

The malaria case estimate keeps the global incidence rate at 57 cases per 1000 population, close to the rate reported since 2014.

Significant progress in reducing malaria incidence includes a 70% decline in the WHO South-East Asia Region (from 17 cases per 1000 population at risk in 2010 to five cases per 1000 population in 2018) and a 22% fall in the WHO African Region (from 294 per 1000 population in 2010 to 229 per 1000 population in 2018).

All other WHO regions recorded either little progress or an increase in incidence.

http://bit.ly/2E0iwmn

## HIV testing guidelines

WHO issued new guidelines on HIV testing to help countries reach the estimated 8.1 million people living with HIV who have yet to be diagnosed.

The guidelines were released on 27 November and make several recommendations, including adoption of a standard HIV testing strategy, which uses three consecutive reactive tests, to improve testing accuracy, self-testing, and social-network-based HIV testing to reach populations at high risk of infection. The guidelines also recommend the delivery of rapid testing through lay providers at community level for relevant countries.

At the end of 2018, an estimated 37.9 million people were living with HIV worldwide. Of these, 79% had been diagnosed, 62% were on treatment and 53% had reduced their HIV levels through sustained treatment.

http://bit.ly/2YBumwH

**Cover photo**

A man enjoys a winter swim in Heilongjiang province, north-east China.

**Figure:**
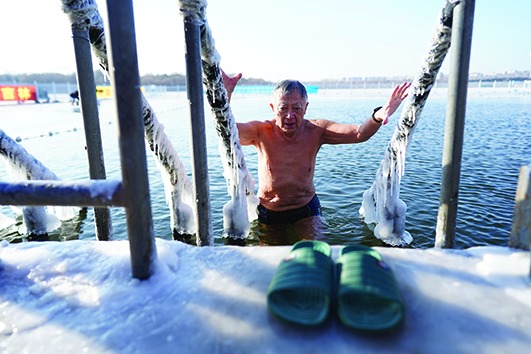
Credit: WHO/Yikun Wang

## Physically inactive children

Some 85% of female and 78% of male from school-going adolescents globally do not meet current recommendations for at least one hour of physical activity per day, according to a new study by WHO experts.

The study, published in *The Lancet Child & Adolescent Health* on 22 November, is based on data reported by 1.6 million 11 to 17-year-olds and is one of the first studies to focus on global trends for adolescent physical activity.

http://bit.ly/2DWrlO6

## Polio eradication pledge.

A group of donors pledged US$ 2.6 billion towards the Global Polio Eradication Initiative’s Polio Endgame Strategy 2019-2023.

The pledge was made at the Reaching the Last Mile Forum in Abu Dhabi on 19 November. It followed an announcement on 24 October, that two of the three wild poliovirus strains have already been eradicated, and expressions of concern regarding polio eradication efforts by the Strategic Advisory Group of Experts (SAGE) on Immunization.

http://bit.ly/2LCRqWD

## Albania earthquake

A 6.4-magnitude earthquake hit Albania on 26 November 2019.

Hundreds of citizens received medical assistance at hospitals in the capital Tirana, as well as in the cities of Durres, Lezha, Kurbin and Kruja. 

WHO staff were deployed to Albania and the WHO country team there conducted a health needs assessment in Durres in collaboration with local and national health officials.

As of 30 November, search-and-rescue operations had ended, and 51 people were confirmed dead by the Albanian health ministry.

http://bit.ly/2P3ajEb

## Iraq and Somalia vaccination campaigns

Health authorities in Iraq, in partnership with WHO and UNICEF, launched a campaign to vaccinate 3.1 million children against polio vaccine in Iraq. The 5-day campaign was launched on 26 November and targeted children in 65 districts in Iraq.

A 5-day campaign in Somalia in November, targeted 1.7 million children under the age of 5 with polio vaccines and children aged 6 to 59 months with measles vaccines.

The campaign was conducted by WHO and UNICEF in partnership with the Somali government.

http://bit.ly/2E65NhP

http://bit.ly/2LDLoVF

**Looking ahead**

26 January. World Leprosy Day

28 January – 2 February. The Prince Mahidol Award Conference. Theme: Accelerating progress towards universal health coverage. Bangkok, Thailand.

3 – 8 February. WHO Executive Board Meeting. WHO Headquarters, Geneva, Switzerland.

